# Emergency Care for Burn Patients—A Single-Center Report

**DOI:** 10.3390/jpm13020238

**Published:** 2023-01-28

**Authors:** Andrei Niculae, Ileana Peride, Mirela Tiglis, Ana Maria Nechita, Lucian Cristian Petcu, Tiberiu Paul Neagu

**Affiliations:** 1 Clinical Department No. 3, “Carol Davila” University of Medicine and Pharmacy, 020021 Bucharest, Romania; 2 Department of Anesthesia and Intensive Care, Emergency Clinical Hospital of Bucharest, 014461 Bucharest, Romania; 3 Department of Nephrology and Dialysis, “St. John” Emergency Hospital, 042122 Bucharest, Romania; 4 Department of Biophysics and Biostatistics, Faculty of Dentistry, “Ovidius” University, 900684 Constanta, Romania; 5 Clinical Department No. 11, “Carol Davila” University of Medicine and Pharmacy, 050474 Bucharest, Romania

**Keywords:** burn injury, epidemiology, regional burn unit, burn etiology, outcome

## Abstract

Burns, one of the main public health problems, lead to significant mortality and morbidity. Epidemiological studies regarding burn patients in Romania are scarce. The aim of this study is to identify the burn etiology, demographics, clinical characteristics, and outcomes in patients requiring treatment in a regional burn unit. Design. We performed a retrospective observational study of 2021. Patients. All patients admitted to our six-bed intensive care unit (ICU) were included. Interventions. The following data were collected for further analysis: demographics, burn pattern (etiology, size, depth, affected body region), type of ventilation, ABSI (Abbreviated Burn Severity Index) score, comorbidities, bioumoral parameters, and hospitalization days. Results. There were 93 burned patients included in our study that were divided into two groups: alive patients’ group (63.4%) and deceased patients’ group (36.6%). The mean age was 55.80 ± 17.16 (SD). There were 65.6% male patients, and 39.8% of the patients were admitted by transfer from another hospital. Further, 59 patients presented third-degree burns, from which 32.3% died. Burns affecting >37% of the total body surface area (TBSA) were noticed in 30 patients. The most vulnerable regions of the body were the trunk (*p* = 0.003), the legs (*p* = 0.004), the neck (*p* = 0.011), and the arms (*p* = 0.020). Inhalation injury was found in 60.2% of the patients. The risk of death in a patient with an ABSI score > 9 points was 72 times higher. Comorbidities were present in 44.1% of the patients. We observed a median LOS (length of stay) of 23 days and an ICU-LOS of 11 days. Logistic regression analysis showed that admission protein, creatinkinase, and leukocytes were independent risk factors for mortality. The general mortality rate was 36.6%. Conclusion. A thermal factor was responsible for the vast majority of burns, 94.6% of cases being accidents. Extensive and full-thickness burns, burns affecting the arms, inhalation injuries, the need for mechanical ventilation, and a high ABSI score represent important risk factors for mortality. Considering the results, it appears that prompt correction of protein, creatinkinase, and leukocytes levels may contribute to improvement in severe burn patients’ outcomes.

## 1. Introduction

Burn injuries can be under-appreciated trauma [1], leading to important mortality and morbidity [2]. Patients presenting with severe or critical burns require rapid specific management, in standardized environments, due to the unique pattern of evolution and complications in the face of an excessive systemic response to injury, with severe hypermetabolic and immunoinflammatory manifestations [3]. Proper care of such patients requires dedicated centers with intensive care units (ICUs), with specialized medical teams being able to offer appropriate resuscitation and monitoring, early and late surgical management, treatment of burn-induced complications, and optimization of previous chronic diseases [3,4,5] in accordance with European Burn Association recommendations and guidelines to standardize burn care around the world [6].

According to the World Health Organization (WHO), burns represent the main public health issue, especially in low- and middle-income countries, being responsible for almost 180.000 deaths per year [7]. Economically, burns are thought to be the most expensive non-lethal injuries [8]. Nevertheless, most importantly, these injuries are considered to produce social stigma due to severe disabilities, disfigurement, and psychological issues. This is more pronounced in women, with a high incidence of post-traumatic stress disorder development [9,10].

Important sources of morbidity and mortality in burn patients are represented by infections and sepsis [11], acute kidney injury [12], acute respiratory failure, especially due to inhalation injury [13], skin sequelae associated with contractures, itching, and chronic pain [14]. As for the psychological component, in addition to the above-mentioned problems, anxiety, depression, negative self-image, and long-term sleep disturbances have a negative impact in the post-burn period [15,16].

Various measures have been taken worldwide to increase public awareness and implement preventive actions, such as installing fire alarms and checking and ensuring a proper number of fire extinguishers in public spaces, including public schools and other educational institutions [17,18]. Therefore, in recent years, there has been a decrease in burn incidence, mortality, and morbidity, especially in developed countries [8,18].

In order to properly and effectively design preventive health care politics and to establish local protocols and funds distribution, understanding regional and national epidemiology of burn injury is required. There are scarce reports about burn management in Romania, although there are few regional burn units. The actual trend is to establish specific burn centers around the country that are aligned with the standards set by the European Burn Society, and we hope that this study may assist in understanding the burden of caring for patients with severe burns. The aim of this retrospective study is to identify the burn etiologies, patterns, demographics, clinical characteristics, and outcomes in patients requiring treatment in a regional burn unit of a level 1 trauma center.

## 2. Materials and Methods

### 2.1. Study Design, Setting, and Population

In this retrospective observational study, conducted between 31 January 2021 and 31 December 2021, all consecutive adult burn patients (age > 18 years) admitted to the Burn Unit of the Emergency Clinical Hospital of Bucharest, Romania were included. Patients were admitted to the ICU with respect to the European Burn Association (EBA) Guidelines for Burn Care regarding the indications for referral to a burn center [6]. As a result of the inconsistent data quality, the study period was limited to one year. There were no exclusion criteria. This study received approval from the Ethical Local Committee. Due to the retrospective type of study, the need for informed consent was waived.

### 2.2. Data Collection

All data were collected using the hospital electronic internal database. Microsoft Excel 2016 (Microsoft) was used to organize the information. Demographic characteristics (age, gender, patients’ environment—urban/rural), burn etiology, type of burn injury (severity), involved body region, total body surface area affected (TBSA%), inflicted injury, inhalation injury, type of ventilation, tracheostomy, days of mechanical ventilation, COVID-19 (coronavirus disease) status, ABSI (Abbreviated Burn Severity Index) score, comorbidities, bioumoral parameters (leukocytes, hemoglobin, thrombocytes, serum total proteins, serum albumin, creatinine, urea, potassium, creatinkinase), length of stay (LOS), and outcome information were obtained from patient registries program. Considering that inappropriate initial management of patients with significant burns can lead to increased injuries depth and more severe burn-shock in the first 24–48 h, with a negative influence on prognosis [19], we considered a transfer as being late if it was performed after 48 h. All personal data were anonymized.

### 2.3. Statistical Analysis

For statistical analysis, we used IBM SPSS statistics software version 23 (IBM Corporation, New York, USA) and MedCalc 14.8.1 (MedCalc Software Ltd., Mariakerke, Belgium). All the data are presented as mean ± standard deviation (SD) for continuous variables in case of symmetric distributions, median, and IQR (interquartile range P75–P25) for continuous variables in case of skewed distributions, or as percentages for categorical variables. The normality of the continuous data was estimated with Kolmogorov–Smirnov test of normality. For hypotheses testing, the following tests were used: independent samples *t*-test, independent samples Mann–Whitney U Test, chi-squared test of association, and *z*-test for comparison of two proportions. Logistic regression was used to find the best fitting model to describe the relationship between the dependent variable (discharge status: deceased/alive) and a set of independent variables (multivariable logistic model). Logistic regression was applied using the backward method and Wald test to test the significance of the coefficients and to observe which parameters are associated with a higher risk of death at time of admission. Following the recommendations of Peduzzi et al. [20], the number of independent variables that can be used was determined:

considering *p*, the smallest of the proportions of deceased or alive patients, and *k*, the number of independent variables, the minimum number of cases that can be included is:

$$N=10\times \frac{k}{p}$$

- our study included 93 patients (N)—59 alive patients (a proportion of 0.634 cases) and 34 deceased patients (a proportion of 0.366 cases); therefore, *p* = 0.366 represents the smallest of the proportions.
- applying the above-mentioned equation, the number of independent variables that can be used in our logistic regression model is *k* = 3.40; in conclusion, maximum 4 variables can be used.

Receiver operating characteristic (ROC) curve analysis was used to establish the threshold value that can distinguish between the two groups (deceased/alive) for the time to reach the endpoint (patient’s death). The significance level α was set at 0.05.

## 3. Results

**Figure 1:**
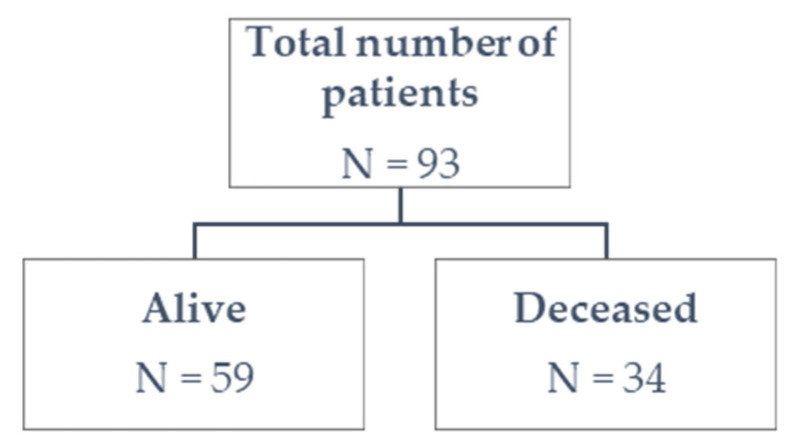
Flowchart of patients from the burn unit from January to December 2021.

From January to December 2021, 93 patients were hospitalized in the six-bed ICU of our regional burn unit and included in this retrospective study (Figure 1). The patients were divided into two groups depending on discharge status: alive patients’ group (63.4%) and deceased patients’ group (36.6%).

### 3.1. General Characteristics

The main characteristics are presented in Table 1. The mean age of all the included subjects was 55.80 ± 17.16 SD 53.58 ± 17.02 SD for the alive patients’ group and 59.68 ± 17.23 SD for the deceased patients’ group). Out of the included patients, 59 survived and 34 died, with no significant differences in mean age (*t* = −1.657, df = 91, *p* = 0.101, 95% CI = −13.410–1.210).

There were *n* = 61 male (65.6%) and *n* = 32 female patients (34.4%) in the general group. We found no association between risk of mortality and patients’ gender (Χ^2^stat = 0.593, df = 1, *p* = 0.441). The risk of death for a man and a woman is the same (OR (odds ratio) = 1.427; 95% confidence interval (CI) = 0.576–3.535).

### 3.2. Primary Hospital Admission, Patients’ Residential Areas, Type, and Timing of Admission

There were 56 patients (60.2%) admitted from the Bucharest area to our emergency department (ED), then into the ICU. The remaining 37 patients (39.8%) arrived in our burn unit by transfer from another hospital (*n* = 23), called “primary hospital” in this study.

**Figure 2:**
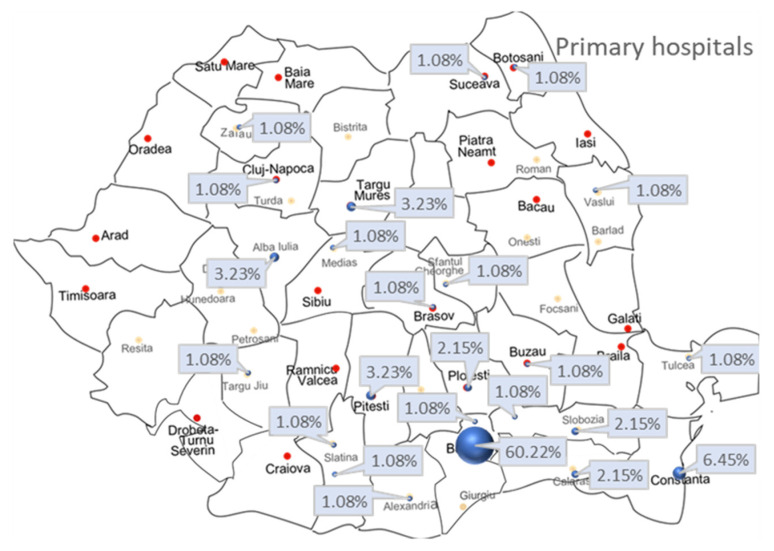
Patients’ distribution according to the primary hospital of admission.

Figure 2 shows the map of Romania and the primary hospital where the patients first arrived after the incident. Each city corresponds to the percentage of patients referred to our burn unit. Due to the shortage of specialized beds to manage such patients, we considered it important to study the timing of admission. Therefore, *n* = 52 (55.9%) patients were admitted directly to our hospital, *n* = 37 (39.8%) patients arrived within 48 h after injury (early transfer), and *n* = 4 (4.3%) patients were transferred after 48 h from incident (late transfer). There was no association between mortality risk and type of admission (direct/early or late transfer) (*p* = 0.218).

**Figure 3:**
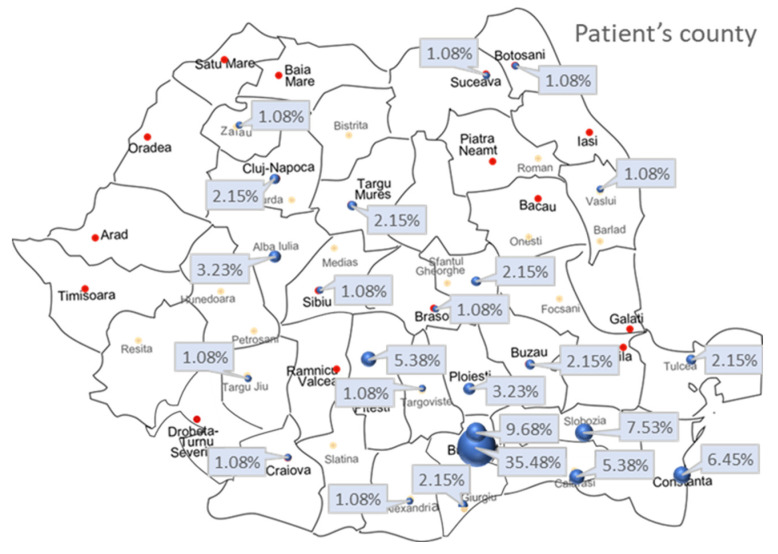
Geographical distribution of patients.

Further, patients’ environment, urban or rural, was recorded (Table 1). Figure 3 shows the map of Romania and the patients’ county of origin.

There was no association between survival status and patients’ environment, rural or urban (Χ^2^stat = 1.431, df = 1, *p* = 0.232). The risk of death for a patient from a rural or an urban environment is the same (OR = 1.682; 95% CI = 0.716–3.953).

### 3.3. Burn Etiology, TBSA (%), Type of Burn (Severity), Inflicted Injury, and Body Region

As can be noticed in Table 1, 29 patients with third-degree burns died (representing 85.3% of the total deceased patients) and 30 survived (50.8% of the total alive patients).

Concerning the burned TBSA (%), *n* = 9 (9.7%) of cases presented critical burns as TBSA > 80% (7.5% of patients with TBSA ≥ 90%). 

To evaluate the difference between burned TBSA (%) in both groups, Mann–Whitney U Test was used: the test revealed significant differences between TBSA% of the deceased group (median = 65%, IQR = 45%, N = 34, mean rank = 69.56) and alive group (median = 20%, IQR = 20%, N = 59, mean rank = 34), *p* < 0.001.

**Figure 4:**
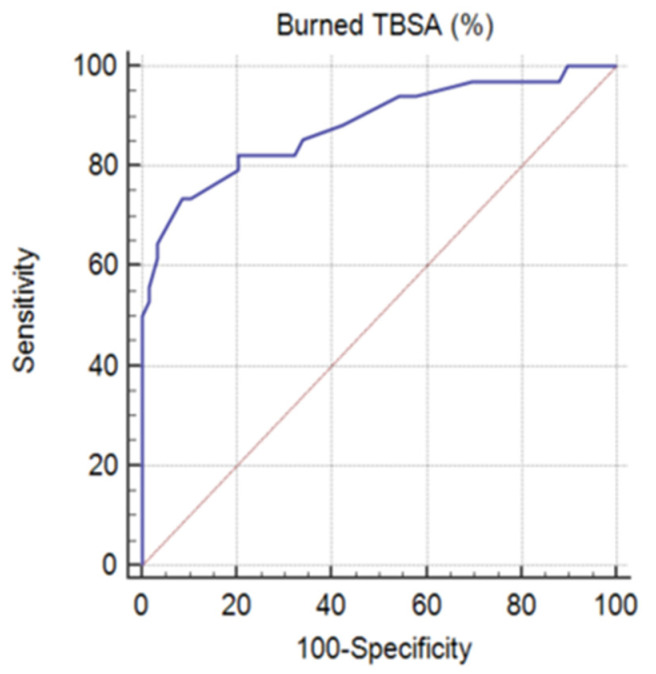
ROC curve for the TBSA (%) variable (TBSA—total body surface area).

Regarding ROC curve analysis (Figure 4), since *p* < 0.001, it can be concluded that the area under the ROC curve is significantly different from 0.5 and the variable burned TBSA (%) has the ability to distinguish between the two groups (A = 0.882, Youden index J = 0.6505, Se = 73.53%, Sp = 91.53%). The threshold value was TBSA > 37%. Therefore, we have further analyzed the influence of this threshold on both groups.

There was an association between mortality risk and TBSA (%) (Χ^2^stat = 41.774, df = 1, *p* < 0.001). The risk of death of a patient when burned TBSA (%) > 37% is 30 times higher than if burned TBSA (%) < 37% (OR = 30; 95% CI = 9.112– 98.771). At the same time, no association was found between mortality risk and mechanism of burn injury (Χ^2^stat = 0.500, df = 1, *p* = 0.480). The risk of death for a patient with thermal injury and a patient with electrocution injury is the same (OR = 0.554; 95% CI = 0.105–2.910).

Another identified issue was related to the type of burn (IIA, IIB, III), which is associated with an increased risk of death (*p* = 0.004). Analyzing the obtained results, with the highest proportion of deceased patients among those with type III burns (85.3% of all patients who died), it is emphasized that, the greater the depth of the burn, the greater the risk of death. Regarding the form of injury, even if it was an accident or self-aggression, there is no association with mortality risk (*p* = 0.870).

In Table 1, we also presented group distribution according to burned body region.

It appears that the most vulnerable regions of the body are the trunk (OR = 4.270; 95% CI = 1.609–11.331), the legs (OR = 3.990; 95% CI = 1.504–10.584), the neck (OR = 3.083; 95% CI = 1.280–7.428), and the arms (OR = 8.426; 95% CI = 1.044–67.983). Importantly, this analysis showed that, in case of arms burn, mortality risk increases 8.426 times.

### 3.4. Inhalation Injury, Type of Ventilation, Tracheostomy, and Days of Mechanical Ventilation

Inhalation injury was found in 38 (40.9%) patients (Table 1). Of the alive patients’ group, *n* = 12 (20.3%) and *n* = 26 (76.5%) patients from the deceased group presented this type of trauma. An association was found between mortality risk and inhalation injury presence (Χ^2^stat = 28.124, df = 1, *p* < 0.001). The risk of death of a patient presenting inhalation injury is 12.729 higher (OR = 12.729; 95% CI = 4.614–35.117).

Regarding type of ventilation, the studied patients were divided into three categories as follows: spontaneous breathing, orotracheal intubation or tracheostomy (early, late), and mechanical ventilation (Table 1).

Type of ventilation is associated with mortality risk (*p* = 0.001), being higher for patients requiring orotracheal intubation and mechanical ventilation. Likewise, presence of tracheostomy appears to be associated with increased risk of mortality (*p* = 0.009).

The distribution of days of mechanical ventilation is not the same across studied categories (alive and deceased groups of patients), with *p* < 0.001 after applying independent samples Mann–Whitney U test. The median value for the deceased patients’ group is 9.5 days, with an IQR of 19 days; meanwhile, the median value for alive patients’ group is 0 (IQR = 2) days. In the alive patients’ group (fifty-nine patients), forty-two patients did not need mechanical ventilation, fifteen patients were ventilated between 1–6 days, and only two patients needed invasive respiratory support for 14 to 20 days. Therefore, the distribution is not symmetric, being skewed (skew = 3707) and leptokurtic (kurt = 16,242), with a heavy tail indicating large outliers. The distribution shape explains the measures of central tendency and dispersion obtained (median (IQR) = 0 (2); mean = 1.47 ± 3.45 SD days).

### 3.5. COVID-19 Status, ABSI Score, Comorbidities, and Bioumoral Parameters

There was no association between positive reverse transcription polymerase chain reaction (RT-PCR) test for severe acute respiratory syndrome coronavirus type 2 (SARS-CoV-2) and risk of death, with a *p* = 0.870. Out of 93 patients, only *n* = 5 (5.4%) were positive, of which *n* = 2 (5.9%) died.

**Figure 5:**
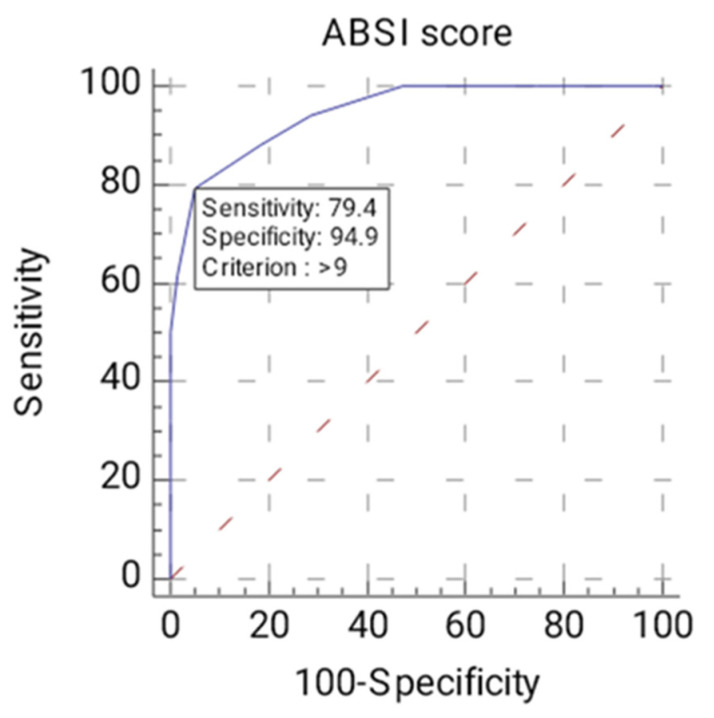
ROC curve for the ABSI variable (ABSI—the Abbreviated Burn Severity Index).

As for the ABSI score, there are statistically significant differences between the mean ABSI scores of deceased patients and those who survived (*t* = 11.020, df = 91, *p* < 0.001). Regarding ROC curve analysis (Figure 5), since *p* < 0.0001, it can be concluded that the area under the ROC curve is significantly different from 0.5 and that there is evidence that the variable ABSI score has the ability to distinguish between the two groups (A = 0.946, Youden index J = 0.7433, Se = 79.41%, Sp = 94.92%). The threshold or criterion value is > 9 points. We further analyzed the influence of the threshold value > 9 on both groups. For ABSI score > 9 points, there are *n* = 27 deceased patients (79.4% of all deceased patients’ group) and *n* = 3 surviving patients (5.1% of all alive patients’ group). As for ABSI score ≤ 9 points, there are *n* = 7 patients who died (20.6% of all deceased patients’ group) and *n* = 56 patients who survived (94.9% of all alive patients’ group). There is an association between the deceased/alive variables and the ABSI score (>9/≤9) (Χ^2^stat = 54.531, df = 1, *p* < 0.001). The risk of death of a patient when ABSI score > 9 points is 72 times higher than if ABSI score ≤ 9 points (OR = 72.00; 95% CI = 17.259–300.366).

In Table 2, we presented the comorbidities encountered among the studied groups.

Further, we have collected information about some bioumoral parameters upon ICU admission and on day 1, as shown in Table 3. 

Taking into consideration burn pathophysiology, the following variables were included: admission—albumin (g/dL), admission—creatinkinase (U/L), admission—leukocytes (val./uL), admission—total proteins (g/dL). Within hours after major burns, there is a dysregulated inflammatory response, proportional to the severity of the injuries and the characteristics of the individual, which can be influenced by initial therapeutic management [21]. Therefore, it is important to present the main bioumoral parameters, both at admission and on day 1 post-injury, considering their influence on patients’ outcome. 

Aside from admission—albumin (g/dL) that was excluded from the model (*p* = 0.131), the other analyzed parameters appear to have a significant impact on risk of mortality (admission—creatinkinase *p* = 0.019; admission – leukocytes *p* = 0.017; admission—total proteins *p* < 0.001). The highly increased values for creatinkinase may be correlated with the presence of severe burns, especially in vulnerable regions, such as legs and arms. 

In Table 4, the adjusted odds ratio (Exp(b)) was described for each of the analyzed parameters (independent variables), which provides the relative amount by which the odds of the outcome (mortality occurrence) increase or decrease in our studied patients. In Table 5, the variable that did not meet the criteria to be included in the regression equation is presented. We observed that the OR for a positive outcome (mortality occurrence) was:

- 1.004 times higher in cases where admission—creatinkinase (U/L) increased by 1 unit, with all other factors remaining unchanged.
- 1.000 times higher in cases where admission—leukocytes (val./uL) increased by 1 unit, with all other factors remaining unchanged.

Regarding admission—total proteins (g/dL), when this parameter increases by 1 unit, with all other factors remaining unchanged, OR will decrease by a factor of Exp(B) = 0.291, meaning the risk of mortality will decrease.

The logistic regression was based on the following equation:

**logit(p) = 2.804 + 0.004∙admission creatinkinase + 0.00012∙admission leukocytes − 1.234∙admission total proteins**

Furthermore, when applying ROC curve analysis, to evaluate the predictive accuracy of the logistic regression model, it was demonstrated that the area under the ROC curve AUC = 0.992 > 0.5 and *p* < 0.001.

### 3.6. Length of Stay and Outcome

Concerning the total days of hospitalization (LOS) and total ICU days of hospitalization (ICU LOS), it was observed that the normal distribution condition is not met. Thus, we introduce the median value and the interquartile range (IQR). For the LOS, it was noted that its distribution within the variables of interest (deceased and alive patients) is not the same, with *p* < 0.001. For the deceased patients’ group, the median (IQR) = 11 (23.75) days. Within the alive patients’ group, the median value (IQR) = 23 (16) days.

The distribution of LOS (days) in the alive patients’ group is the same across categories of TBSA (≤37%, >37%), *p* = 0.843 (independent samples Mann–Whitney U Test). For the deceased patients’ group, overall, the LOS median was 11 days (IQR = 23.75 days), and, for the alive patients’ group, it was 23 days (IQR = 16 days).

**Figure 6:**
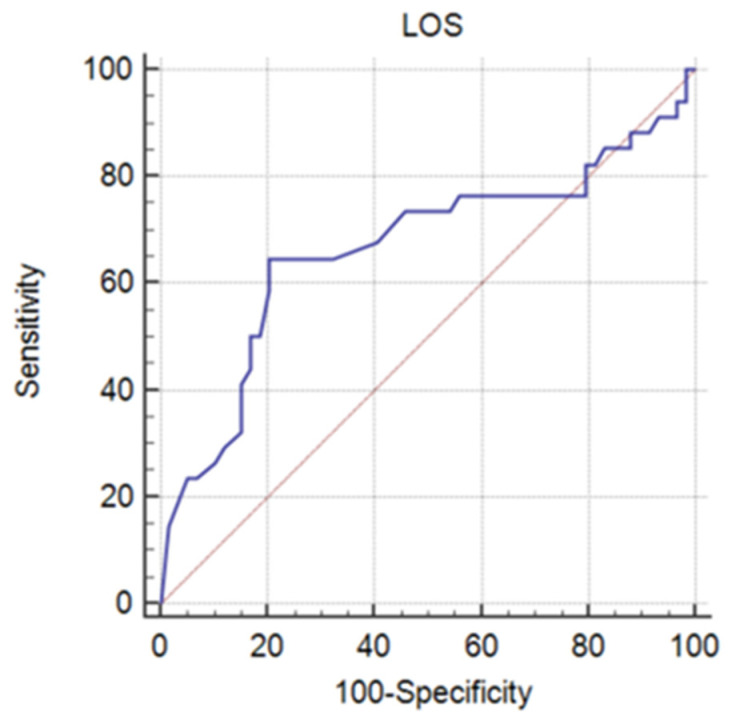
ROC curve for the length of hospitalization (LOS) variable.

ROC curve analysis (Figure 6) was used to establish the threshold value that can distinguish between the two studied groups for the time to reach the endpoint (patient’s death). Since *p* = 0.0104, it can be concluded that the variable LOS (days) has the ability to distinguish between the two groups (A = 0.666, Youden index J = 0.4437, Se = 64.71%, Sp = 79.66%). The threshold value for LOS was ≤ 14 days.

For the ICU LOS, it is observed that its distribution within the variables of interest (deceased and alive patients) is not the same, with *p* < 0.001. Regarding the deceased patients’ group, the median value was 11 days, with IQR of 23.75 days. As for the alive patients’ group, the median value (IQR) was 18 (21). Considering that *p* = 0.2976, the area under the ROC curve is not significantly different, and, therefore, the variable ICU LOS (days) does not have the ability to distinguish between the two studied groups.

Further, we analyzed the influence of the threshold or criterion value ≤ 14 days on both groups. In the case of LOS ≤ 14 days, there are *n* = 22 deceased patients (64.7% of the deceased patients’ group) and *n* = 12 alive patients (20.3% of the alive patients’ group). As for LOS > 14 days, *n* = 12 deceased patients (35.3% of the deceased group patients) and *n* = 47 alive patients (79.7% of the alive patients’ group). We found an association between deceased/alive categorical variables and LOS (days) (≤14/>14) (Χ^2^stat = 18.306, df = 1, *p* < 0.001). The risk of death of a patient when LOS ≤ 14 days is 7.181 higher than if LOS > 14 days (OR = 7.181; 95% CI = 2.786–18.509).

All patients presenting critical burns (TBSA ≥ 80%) died. Among the studied groups, the general mortality rate was 36.6%, with the groups’ distribution presented in Figure 1.

**Figure 7:**
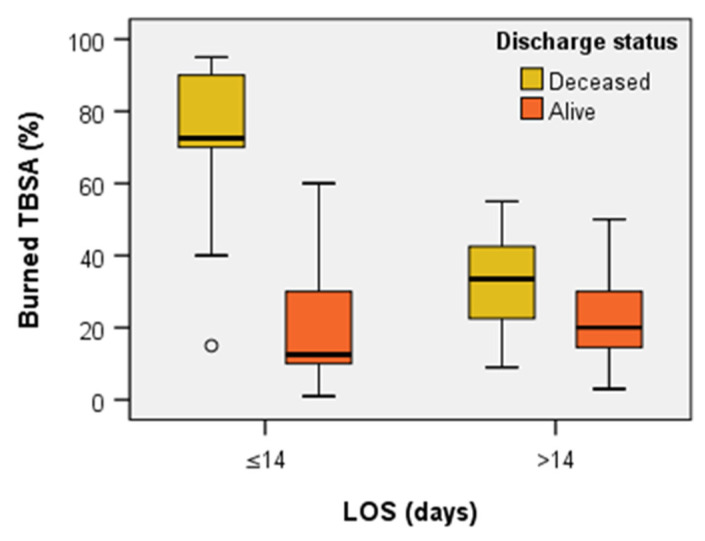
Comparison of TBSA (%) with LOS and patients’ discharge status. TBSA—total body surface area; LOS—length of stay.

The Mann–Whitney U Test revealed significant differences in TBSA (%) for LOS ≤ 14 days between the deceased patients’ group (median = 72.50, *n* = 22, mean rank = 23.09) and alive patients’ group (median = 12.50, *n* = 12, mean rank = 7.25), U= 9.00, z = −4.457, *p* < 0.001 and also significant differences in TBSA (%) for LOS > 14 days between the deceased patients’ group (median = 33.50, *n* = 12, mean rank = 40.83) and alive patients’ group (median = 20.00, *n* = 47, mean rank = 27.23), U = 152.00, z = −2.458, *p* = 0.014 – Figure 7. Summarizing, we noticed that the burned area of the deceased patients was greater than that of the surviving ones, especially in the first 14 days.

## 4. Discussion

We present a report about the epidemiology, burn pattern, and mortality risk factors from a burn unit in Romania. In our country, there are important burn units in Bucharest, Timisoara, and Iasi (24 ICU beds for adult patients) [22]. As presented in Figure 2, irrespective of the hospital to which patients with severe burn were first referred, in our unit, there were admitted patients from all over the country, except for the western area served mainly by the burn care unit from Timisoara and the eastern area served by the burn care unit from Iasi. An epidemiological study by Pieptu et al. analyzed all the burned patients hospitalized in Romania—children and adults—according to the Center of National School of Public Health, Management and Professional Development database irrespective of burn severity [23]. In contrast, the present analysis targets the adult subpopulation presenting with burns requiring acute specific intensive care management, patients who, according to international criteria, entail transfer and care in a specialized burn center/unit [6]. Thus, a strength of this study is that it carries out an analysis of patients with major burns, which has not been studied in detail to date in Romania. In comparison with the above-mentioned study [23], we observed a higher incidence of cases from urban areas, a longer LOS for the alive patients’ group, and a higher mortality rate. The same pattern was noticed with respect to burn incidence among men.

In many studies, as in ours, men are more often affected by severe burns and require hospitalization, perhaps due to high-risk occupations, an aspect reported especially in low- and middle-income countries [24,25]. This trend is reported in Brazil [26], Europe [2,24], and Colombia [27], but not in India, with a 1.7:1 female:male ratio [28], and there is a slightly more common female predominance in Nepal [29].

The mean age in our study was 55.8 years, much higher than other reports, such as 30.6 years [29], 27.0 years [30], 24.9 years [31], or a median age of 38 (IQR:28–52) [32] or 22 years (IQR:2–53) [33]. It can be a consequence of the fact that our burn unit is exclusively for adult patients, unlike most reports.

Thermal burns are the most common in this study (over 90% of cases), in line with previous reports, including developing countries [28,29,34]. Staten Island, New York, showed that scalding injuries were regularly observed (59% of cases), as well as in Southwest China, followed by contact injury or flame [30,33]. The second burn mechanism was electrocution (around 6%), different from South India, Nepal, or Iraq with scald injuries [28,29,35].

Regarding the form of injury, in our study, around 94.6% were accidents and only 5.4% self-aggressions, without significant correlation with the outcome. Ganesamoni et al. reported a rate of 52.5% accidental burns and 43.9% injuries resulted from self-immolation, especially among women [28]. A report from Brazil showed that 78.8% of patients were victims of various accidents, 11.9% of cases were self-aggressions, and 9.2% were victims of attempted murders [32].

In the present analysis, the most vulnerable regions of the body are, in order of importance, the trunk, the legs, the neck, and the arms. Fan et al. published a study involving 3376 burned patients showing that, for all age groups, the face, trunk, and extremities are frequently the most hurt parts [36]. Karki et al. reported that lower (40%) and upper (32.2%) extremities were generally affected [29]. Hanh et al. showed that, in a regional burn unit from Staten Island, New York, the lower extremities suffered the most (29% of cases) [33]. In a study from Southwest China, limbs were commonly involved (72.1%), followed by the head, face, and neck region (47.7%), then the trunk (43.9%) [30].

Third-degree burns were found in 63.4% of cases in the present analysis, different from other studies in which ≥50% of patients presented second degree burns [33,35]. A report from Albania showed that full-thickness burns affected around 17% of patients [31], Galicia (Spain) reported 9.5% of cases [37], Pakistan 33% [38], and China 40.1% [30]. This may be a consequence of accumulation of a higher number of severe burn cases per care unit due to the limited number of intensive care beds nationwide dedicated to care of these patients.

TBSA (%), the threshold value, above which mortality risk increases, was found to be 37%. Various studies have similar results, with TBSA >40% [28] or >35% [39] being a risk factor for a negative outcome. However, there are many reports showing lower percentages of patients with extensive burns, such as Pegg reporting only 3% of cases with TBSA of 41–60% [40], Lami and Naser with around 16% of patients with >40% TBSA burned [35], and Gilbert et al. with 22.4% of cases presenting burned TBSA > 10% [41].

Although there is a study reporting that presence of inhalation injury is not a predictor of poor outcome, it indicates that none of the patients presenting with it survived [28]. In the present analysis, the risk of mortality is 12.729 higher in the presence of this trauma than without it. This is in line with various other reports showing that patients with inhalation injuries have increased risk of death [33,42]. Bagheri et al. reported that this trauma is associated with amount of required surgical interventions, predicts need for intubation, and significantly influences mortality [42]. As for mechanical ventilation, irrespective of the presence or absence of inhalation injuries, it was required in 50.5% of cases. Other studies report a rate of 45.7% [32] or 6.8% [37]. Tracheostomy was performed in 7.5% of patients, lower than other reports, such as 15.7% [32].

Comorbidities were found in 44.1% of cases. There are reports with different results, varying from 5.2% [28] to values similar to our analysis, such as 40% [43]. Things become much more complicated when comorbidities appear at younger ages [43]. A recent study focused on impact of comorbidities on clinical outcome of burn patients. The authors showed that preexisting comorbidities, such as chronic obstructive pulmonary disease (COPD) and renal insufficiency, have a significant negative impact on severe burned patients’ evolution. In the presence of a single comorbidity, renal insufficiency has been shown to prolong hospitalization [44]. Brandão et al. suggested that comorbidities should be included in burn admission scores because we will be able to better predict mortality in this population [45].

Over the years, various scoring systems have been proposed to assess outcomes in patients with significant burns. Therefore, cut-off values to establish a correlation with mortality risk should be adapted in each unit. We currently use ABSI score according to our internal protocols. In our case, the risk of death of a patient when the ABSI score is > 9 points is 72 times higher than when the score is lower. In a report by Li et al., ABSI was also considered to be more suitable in predicting a negative outcome compared with Baux or PBI (Prognostic Burn Index) scores, with a higher AUC and the smoothest ROC [30]. Queiroz et al. found a cut-off point of 7 points to discriminate between survivors and deceased (78% sensitivity, 84.9% specificity) [32].

We identified a median value for LOS of 23 days and a median ICU-LOS of 11 days. Li et al. showed a median LOS of 17 days [30]. A report from Brazil presented a median ICU-LOS of 12 (IQR:6–23) and median LOS of 21 (IQR:14–33) [32]. Palacios García et al. showed an average LOS of 15.55 days [37].

Considering the main factors involved in morbidity and mortality burden in burn patients, we have chosen to use specific markers, of which albumin, total protein, creatinkinase, leukocytes, and urea value at admission and on day one (but also potassium value on day one) were associated with mortality risk. A recent study by Bandeira et al. showed that low serum albumin concentration at admission increases morbidity and mortality of burned patients [46]. As already known, acute kidney injury is an independent risk factor for mortality regarding severe burns [47]. Emami et al., in a cohort of 258 patients with burns, reported that blood urea nitrogen was independently associated with acute kidney injury development [48]. Regarding our cohort, extending the monitoring period for bioumoral markers throughout hospitalization will enable identifying predictive values.

We have used logistic regression to identify parameters responsible for increasing mortality risk. Knowing the burn pathophysiology, with tissue damage, hyperpermeability, protein leak, capillary leak syndrome responsible for polycompartment syndrome, and high risk of infection when the burned TBSA is severely affected, we have chosen total protein, albumin, creatinkinase, and leukocytes for further analysis [1,11,49]. Aside from albumin, the other biomarkers seemed to be independent risk factors for mortality.

The overall mortality rate was 36.6% in our burn unit (70.6% in males and 29.4% in females). The fact that almost 10% of patients presented with critical burns (TBSA > 80%) was a driving factor, along with the fact that the majority of the studies present overall mortality, including patients with limited burn injuries or children with high survival rates. In Sri Lanka, burns involving >50% TBSA were associated with fatal prognosis [50]. Other reports about patients with critical burns show mortality rates as high as 60.8% (71.4% in females and 46% in males) [28] or 100% [50]. This is a trend frequently encountered in important referral hospitals in low- and middle-income countries [28]. However, there are developed countries reporting mortality rates of 1.2% (Spain) [37], around 3% (United States, Netherlands, Sweden) [51,52,53], 5.4% (Finland) [54], or developing countries with 13.3% (Iraq) [35] or 14% (Pakistan) [38]. A study from Albania showed a mortality rate of 1.05% for children, 7.91% for adults, and 26.58% for elderly patients [31]. Brazil determined a mortality rate at hospital discharge of 34.1% [32].

The risk factors for mortality in our patients were: TBSA burned >37% (over this value, mortality risk increases 30 times), burn depth (the grater the depth of the burn, the greater the mortality risk), presence of inhalation injury (12.729 times higher risk of negative outcome), requirement of orotracheal intubation or tracheostomy, ABSI score > 9 points (72 times higher mortality risk), burns affecting arms (8.426 times higher risk), and increased level of creatinkinase and leukocytes at admission. Reports show that, in almost 80% of severe burn cases, the arms are affected, especially due to protective reflexes, and, as observed in this study, there was an increased percentage of patients with third-degree burns and extended TBSA% burned [55]. Interestingly, an LOS of ≤14 days was associated with a 7.181 higher risk of mortality, translating the severity of cases cared for in our burn unit. Second, we discovered that increasing the value of total proteins at admission with one unit can decrease mortality risk, a variable that we can act on in the future.

The first limitation of this study is the impossibility to compare results with the regional situation due to the lack of reports. Second, our burn unit received patients with critical burns transferred from other hospitals, increasing the burn burden and mortality rate. Third, this study included data from a single unit, and it is hard to know how much the data can be extrapolated to the general population. Another limitation is represented by the fact that the bioumoral parameters are limited due to retrospective data inconsistency, and they were not monitored during the entire hospitalization to analyze their prognostic performance in terms of LOS or mortality. Moreover, the small sample size reduces the power of the findings.

## 5. Conclusions

This study presents the epidemiology, burn injury pattern, and outcomes in a major burn unit from Romania. A thermal factor was responsible for the vast majority of cases; therefore, preventive measures and population-based measures can be planned. Extensive and full-thickness burns, burns affecting the arms, the presence of inhalation injury, the need for mechanical ventilation, and high ABSI scores represent the main risk factors for mortality. Interventional therapeutic measurements directed to improve protein, creatinkinase, and leukocytes levels appear to be beneficial in improving severe burn patients’ outcome.

## Figures and Tables

**Table 1:** Characteristics and distribution of the studied population.

Characteristics			Alive Patients’ Group	Deceased Patients’ Group	Total (N; %)	*p*-Value
Age (mean ± SD, years)		Female	60.50 ± 17.81	62.60 ± 14.46	32 (34.4%)	0.746
		Male	49.46 ± 15.32	58.46 ± 18.40	61 (65.6%)	0.043
Gender (N; %)		Female	22 (37.3%)	10 (29.4%)	32 (34.4%)	0.585
		Male	37 (62.7%)	24 (70.6%)	61 (65.6%)	0.585
Patients’ environment		Urban (N; %)	37 (62.7%)	17 (50%)	54 (58.1%)	0.328
		Rural (N; %)	22 (37.3%)	17 (50%)	39 (41.9%)	0.328
Variables	Mechanism of injury (N; %)	Thermal	56 (94.9%)	31 (91.2%)	87 (93.5%)	0.793
		Electric	3 (5.1%)	3 (8.8%)	6 (6.5%)	0.739
	TBSA (%)	>37%	5 (8.5%)	25 (73.5%)	30 (32.3%)	<0.001
		≤37%	54 (91.5%)	9 (26.5%)	63 (67.7%)	<0.001
	Type of burn (degree)	IIA	4 (6.8%)	1 (2.9%)	5 (5.4%)	0.744
		IIB	25 (42.4%)	4 (11.8%)	29 (31.2%)	0.004
		III	30 (50.8%)	29 (85.3%)	59 (63.4%)	0.001
	Form of injury	Accident	56 (94.9%)	32 (94.1%)	88 (94.6%)	0.754
		Self-aggression	3 (5.1%)	2 (5.9%)	5 (5.4%)	0.754
Body region		Head	44 (74.6%)	27 (79.4%)	71 (76.3%)	0.597
		Neck	22 (37.3%)	22 (64.7%)	44 (47.3%)	0.011
		Trunk	28 (47.5%)	27 (79.4%)	55 (59.1%)	0.003
		Abdomen	21 (35.6%)	16 (47.1%)	37 (39.8%)	0.277
		Pelvic	24 (40.7%)	18 (52.9%)	42 (45.2%)	0.252
		Arms	47 (79.7%)	33 (97.1%)	80 (86.0%)	0.020
		Legs	12 (20.3%)	26 (76.5%)	38 (40.9%)	0.004
Type of ventilation		Spontaneous breathing	42 (71.2%)	4 (11.8%)	46 (49.5%)	<0.001
		Orotracheal intubation	16 (27.1%)	24 (70.6%)	40 (43.0%)	<0.001
		Tracheostomy early < 48 h late > 48 h	1 (1.7%) 1 (1.7%) 0 (0%)	6 (17.6%) 1 (2.9%) 5 (14.7%)	7 (7.5%) 2 (2.2%) 5 (5.4%)	0.016 0.719 0.010
Positive RT-PCR for SARS-CoV-2			3 (5.1%)	2 (5.9%)	5 (5.4%)	0.870
Inhalational injury		yes no	12 (20.3%) 47 (79.7%)	26 (76.5%) 8 (23.5%)	38 (40.9%) 55 (59.1%)	<0.001 0.001
ABSI score (mean ± SD)			6.59 ± 1.81	11.32 ± 2.28	NA	<0.001
LOS (median, IQR; days)			23 (16)	11 (23.75)	NA	0.008
ICU-LOS (median, IQR; days)			18 (21)	11 (23.75)	NA	0.276

Note: TBSA—total body surface area; RT-PCR—reverse transcription polymerase chain reaction; SARS-CoV-2—severe acute respiratory syndrome coronavirus type 2; ABSI—the Abbreviated Burn Severity Index; LOS—length of stay; ICU-LOS—intensive care unit length of stay; NA—not applicable; SD—standard deviation; IQR—interquartile range.

**Table 2:** Patients’ distribution according to present comorbidities.

Comorbidity	Alive Patients’ Group (N = 59; %)	Deceased Patients’ Group (N = 34; %)	*p*-Value
Without	34 (57.6%)	18 (52.9%)	0.823
Cardiac	7 (11.9%)	3 (8.8%)	0.906
Metabolic	7 (11.9%)	2 (5.9%)	0.563
Respiratory	2 (3.4%)	0 (0%)	0.729
Myasthenia gravis	0 (0%)	1 (2.9%)	0.791
Polyarthritis	0 (0%)	1 (2.9%)	0.791
Neurological	0 (0%)	1 (2.9%)	0.791
Vascular	1 (1.7%)	0 (0%)	0.781
Trauma	2 (5.9%)	2 (3.4%)	0.969
Infection	1 (1.7%)	0 (0%)	0.781
Psychiatric	1 (1.7%)	0 (0%)	0.781
Mixed	8 (13.6%)	6 (17.6%)	0.827

**Table 3:** Comparative analysis of laboratory parameters between the studied groups.

Parameter	Alive Patients’ Group		Deceased Patients’ Group		*p*-Value
	median	IQR	median	IQR	
Admission—albumin (g/dL)	2.53	1.57	3.98	0.96	<0.001
Day 1—albumin (g/dL)	1.84	0.87	3.20	1.20	<0.001
Admission—creatinkinase (U/L)	310.50	1138.19	144.70	148	<0.001
Day 1—creatinkinase (U/L)	343.59	1996.62	129	102	<0.001
Admission—creatinine (mg/dL)	0.96	0.55	0.81	0.29	0.092
Day 1—creatinine (mg/dL)	0.77	0.36	0.71	0.22	0.133
Admission—leukocytes (val./uL)	19,480	12,700	11,470	7910	<0.001
Day 1—leukocytes (val./uL)	14,800	11,875	11,170	6970	0.032
Admission—hemoglobin (g/dL)	15.95	4.59	15.20	2.2	0.174
Day 1—hemoglobin (g/dL)	13.77	4.88	13.60	2.9	0.895
Admission—thrombocytes (val./uL)	284,500	244,750	224,000	86,600	0.860
Day 1—thrombocytes (val./uL)	171,450	154,650	204,000	82,000	0.137
Admission—potassium (mmol/L)	4.06	1.46	4.07	0.63	0.917
Day 1—potassium (mmol/L)	4.37	1.20	4.12	0.60	0.005
Admission—total proteins (g/dL)	4.35	1.44	5.97	1.40	<0.001
Day 1—total proteins (g/dL)	3.43	1.43	5.10	1.26	<0.001
Admission—urea (mg/dL)	40.35	38.77	32.50	14.50	0.028
Day 1—urea (mg/dL)	32.65	40.80	27.80	17.60	0.035

Note: IQR—interquartile range P75–P25.

**Table 4:** The influence of bioumoral parameters on the relative instantaneous risks of mortality in our patients (variables included in the regression equation).

Variables	B	Sig.	Exp(B)
Admission—creatinkinase (U/L)	0.004	0.019	1.004
Admission—leukocytes (val./uL)	0.000127	0.017	1.000
Admission—total proteins (g/dL)	−1.234	0.000	0.291
Constant	2.804	0.153	16.518

Note: the table lists the variables included in the model, their regression coefficient B, and Exp(B). This factor Exp(B) is the odds ratio (OR) for the independent variable, and it provides the relative amount by which the odds of the outcome increase (OR greater than 1) or decrease (OR less than 1) when the value of the independent variable is increased by 1 unit.

**Table 5:** Variable not included in the regression equation.

Variables	Score	df	Sig.
Admission—albumin (U/L)	2.277	1	0.131

## Data Availability

Database is available, upon reasonable request, to the corresponding authors.
